# Randomised trials of temperature management in cardiac arrest: Are we observing the Zeno’s paradox of the Tortoise and Achilles?

**DOI:** 10.1186/s13054-021-03826-9

**Published:** 2021-11-27

**Authors:** Tommaso Scquizzato, Paul J. Young, Giovanni Landoni, Luisa Zaraca, Alberto Zangrillo

**Affiliations:** 1 Department of Anesthesia and Intensive Care, IRCCS San Raffaele Scientific Institute, Via Olgettina, 60, 20132 Milan, Italy; 2 Medical Research Institute of New Zealand, Wellington, New Zealand; 3 Faculty of Medicine, Vita-Salute San Raffaele University, Milan, Italy

## To the Editor,

Initial trials published in 2002 found a benefit of therapeutic hypothermia at 32–34 °C in comatose adults resuscitated after cardiac arrest [1, 2]. Two decades after the publication of these practice-changing trials, a large multicentre randomised clinical trial (mRCT) found no benefit of temperature control at 33 °C compared to normothermia with active treatment of fever [3].

mRCTs performed in critically ill patients frequently do not confirm the positive findings of smaller or single-centre RCTs (sRCTs) [4]. Early positive studies on hypothermia had many methodological shortcomings, including no power calculations, small samples, unblinded assessors, nonstandard neuro-prognostication, and discontinuation because of funding lack. Moreover, the control group experienced fever, and it was unclear if improved outcomes were attributable to hypothermia or fever avoidance. Therefore, beneficial effects of hypothermia might be overestimated or could represent type 1 errors. In contrast, recent studies had lower risk of bias, larger samples, and higher methodological quality. The TTM2 trial enrolled five times more patients than earlier trials combined, minimised premature withdrawal of care, applied rigorous prognostication guidelines, protocolised care, and actively treated fever in both groups [3, 5].

Are we observing the Zeno’s paradox of the Tortoise and Achilles? In this paradox, Achilles is racing with a Tortoise with a head start. According to Zeno, Achilles will never reach the Tortoise, as every time Achilles reaches where the Tortoise was, the Tortoise moved forwards. Are the earlier, small, often sRCTs on temperature management the Tortoise and the recent, large, mRCTs Achilles? The more powerful mRCTs (Achilles) continuously chase sRCTs (Tortoise), but they cannot reach them and always arrive later, often with different findings and unavoidable delay. Since the publication of earlier studies, treatments improved (i.e. coronary angiography, standardised haemodynamic/ventilatory targets, early withdrawal avoidance) and may have influenced intervention effects. Moreover, differences between treatment and control are progressively muffling due to parallel treatments competing with the studied intervention. Consequently, patients needed to enrol and the power is never sufficient.

The Zeno’s paradox may initially reflect the clinical trials reality where mRCTs (Achilles) never reach sRCTs (Tortoise). However, it does not, just like in the real world where Achilles can reach the Tortoise. Slowly and painfully, mRCTs can reach and confirm the findings of sRCTs and, when it occurs, worldwide clinical practice changes. Until that happens, the positive findings of sRTCs should be interpreted cautiously, unless confirmed by high-quality mRCTs, particularly when such studies are unblinded.

## Data Availability

Not applicable.

## Abbreviations

mRCT: Multicentre randomised clinical trial
sRCT: Single-centre randomised clinical trial
TTM2: Targeted hypothermia versus targeted normothermia after out-of-hospital cardiac arrest
